# Tuning Myogenesis by Controlling Gelatin Hydrogel Properties through Hydrogen Peroxide-Mediated Cross-Linking and Degradation

**DOI:** 10.3390/gels8060387

**Published:** 2022-06-17

**Authors:** Wildan Mubarok, Kelum Chamara Manoj Lakmal Elvitigala, Shinji Sakai

**Affiliations:** Department of Materials Engineering Science, Graduate School of Engineering Science, Osaka University, Toyonaka 560-8531, Japan; wildanmubarok@cheng.es.osaka-u.ac.jp (W.M.); kelum@cheng.es.osaka-u.ac.jp (K.C.M.L.E.)

**Keywords:** horseradish peroxidase, myoblast, skeletal muscle, tissue engineering, gelatin

## Abstract

Engineering skeletal muscle tissue in vitro is important to study the mechanism of myogenesis, which is crucial for regenerating muscle cells. The physicochemical properties of the cellular microenvironment are known to govern various cell behaviours. Yet, most studies utilised synthetic materials to model the extracellular matrix that suffers from cytotoxicity to the cells. We have previously reported that the physicochemical property of hydrogels obtained from horseradish peroxidase (HRP)-catalysed cross-linking could be controlled by a simple adjustment to the exposure time to air containing H_2_O_2_. In this study, we evaluated the influence of physicochemical properties dynamics in the gelatin possessing phenol groups (Gelatin-Ph) hydrogel to regulate the myogenesis in vitro. We controlled the Young’s modulus of the Gelatin-Ph hydrogel by tuning the air containing 16 ppm H_2_O_2_ exposure time for 15–60 min. Additionally, prolonged exposure to air containing H_2_O_2_ also induced Gelatin-Ph degradation. Myoblasts showed higher adhesion and myotube formation on stiff hydrogel (3.53 kPa) fabricated through 30 min of exposure to air containing H_2_O_2_ compared to those on softer hydrogel (0.77–2.79 kPa) fabricated through 15, 45, and 60 min of the exposure. These results demonstrate that the myogenesis can be tuned by changes in the physicochemical properties of Gelatin-Ph hydrogel mediated by H_2_O_2_.

## 1. Introduction

Skeletal muscle is the largest component of the human body, accounting for 30–40% of body mass [1,2]. In the occurrence of traumatic injuries or degenerative diseases, skeletal muscle can be damaged, which causes a physiological impairment. To rescue the physiological function, the formation of new muscle cells (myogenesis) is needed. While native cells could regenerate the muscle tissue, controlling the fate of these cells to differentiate into muscle cells is difficult due to the complex interaction between the intrinsic factors of the cells and external factors, including their microenvironment. The tissue engineering approach has garnered great interest since it provides an in vitro model to study the physiological phenomenon regulating myogenesis that could improve the efficiency of cell therapy [3,4].

Among the important factors that govern myogenesis are the materials comprising the extracellular matrix (ECM) and the physicochemical properties of the ECM. To study the physicochemical effect of the substrate on myoblasts, most studies utilised synthetic materials such as poly(ethylene glycol) diacrylate (PEGDA), poly(vinyl alcohol) (PVA) gels, and poly(acrylamide) [5,6]. Zahari et al. utilised laminin-coated poly(methyl methacrylate) nanofiber scaffold [7], while Shin et al. reported the use of poly(lactic-co-glycolic acid) [8]. Additionally, the graphene oxide-based matrix is also widely used to control myoblast differentiation [9,10]. However, these materials are not components of natural ECM and suffer from the toxicity of the uncross-linked monomers and photo-crosslinkers [5,6].

To model the native ECM environment, gelatin is widely used due to its excellent biocompatibility and biodegradability [11]. In recent years, several studies have reported the application of gelatin-based hydrogels to control myoblasts’ behaviour. Hayashi et al. reported gelatin-conjugated supramolecular hydrogels with a switchable stiffness [12]. While C2C12 adhesion showed dynamic changes according to the stiffness, this study does not report the myoblasts differentiation. Denes et al. fabricated micropatterned gelatin hydrogels to study the myotube orientation [13]. Du et al. also successfully achieved directed cell migration and myotube formation using 3D-printed gelatin methacrolyl (GelMA) micropatterns on a surface coated with thermo-responsive material poly(*N*-isopropylacrylamide) [14]. However, these studies do not consider the effect of the physicochemical properties of the gelatin hydrogel, which also play a key role in myogenesis.

**Figure 1:**
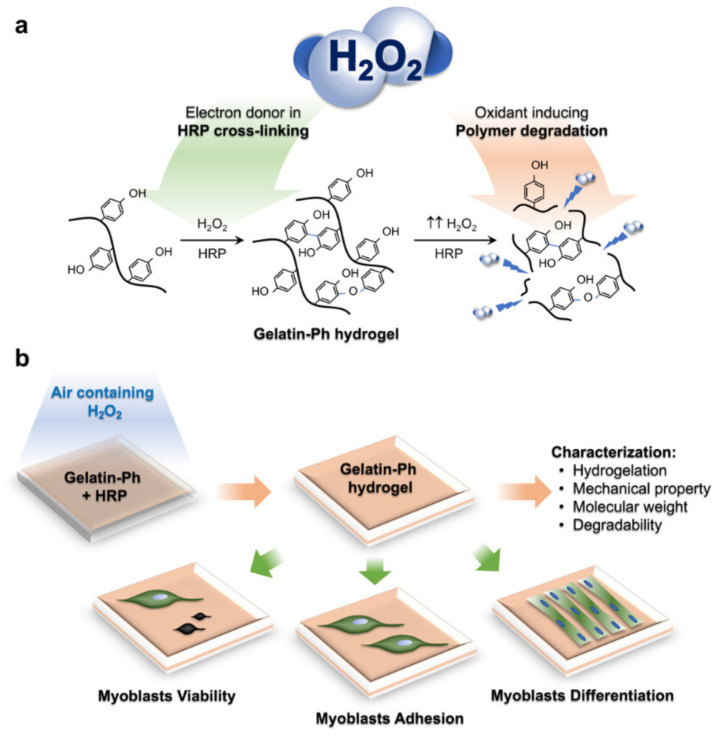
(**a**) The conceptual scheme of the contradictory function of hydrogen peroxide (H_2_O_2_) to induce horseradish peroxidase (HRP)-catalysed cross-linking and polymer degradation of the Gelatin-Ph. (**b**) Experimental scheme of this study. Gelatin-Ph hydrogels fabricated by tuning air containing H_2_O_2_ exposure time were characterised, and the effect on myoblasts’ viability, adhesion, and differentiation was studied.

Recently, we have reported that hydrogen peroxide (H_2_O_2_) could be used to control the physicochemical property of gelatin derivatives possessing phenolic hydroxyl moieties (Gelatin-Ph) hydrogel obtained from horseradish peroxidase (HRP)-catalysed cross-linking [15]. This system exploits the contradictory effect of H_2_O_2_ that simultaneously induces the HRP-catalysed cross-linking as an electron donor while degrading the polymer as an oxidant (Figure 1a) [15]. The advantage of this system is that the mechanical property and molecular weight of the hydrogel can be controlled by a simple adjustment of the air containing H_2_O_2_ exposure time. Using this system, the adhesion of stem cells and fibroblasts can be controlled [15]. However, there are no reports that study the effect of the dynamics of the physicochemical properties of the Gelatin-Ph hydrogel by H_2_O_2_ to control the myogenesis.

In this study, we aimed to investigate the effect of the H_2_O_2_–mediated control of the physicochemical properties of Gelatin-Ph on modulating myogenesis. To address this, we fabricated the Gelatin-Ph hydrogel by exposing air containing H_2_O_2_ at different exposure times, and the hydrogel properties were characterised. The modulatory effect of these physicochemical changes of Gelatin-Ph on myoblast behaviour is studied to the myoblast adhesion and viability. In addition, we also report the influence of the H_2_O_2_ contradicting effect on the formation of myotubes on the Gelatin-Ph hydrogel (Figure 1b).

## 2. Results and Discussion

### 2.1. Gelatin-Ph Hydrogel Characterisation

**Figure 2:**
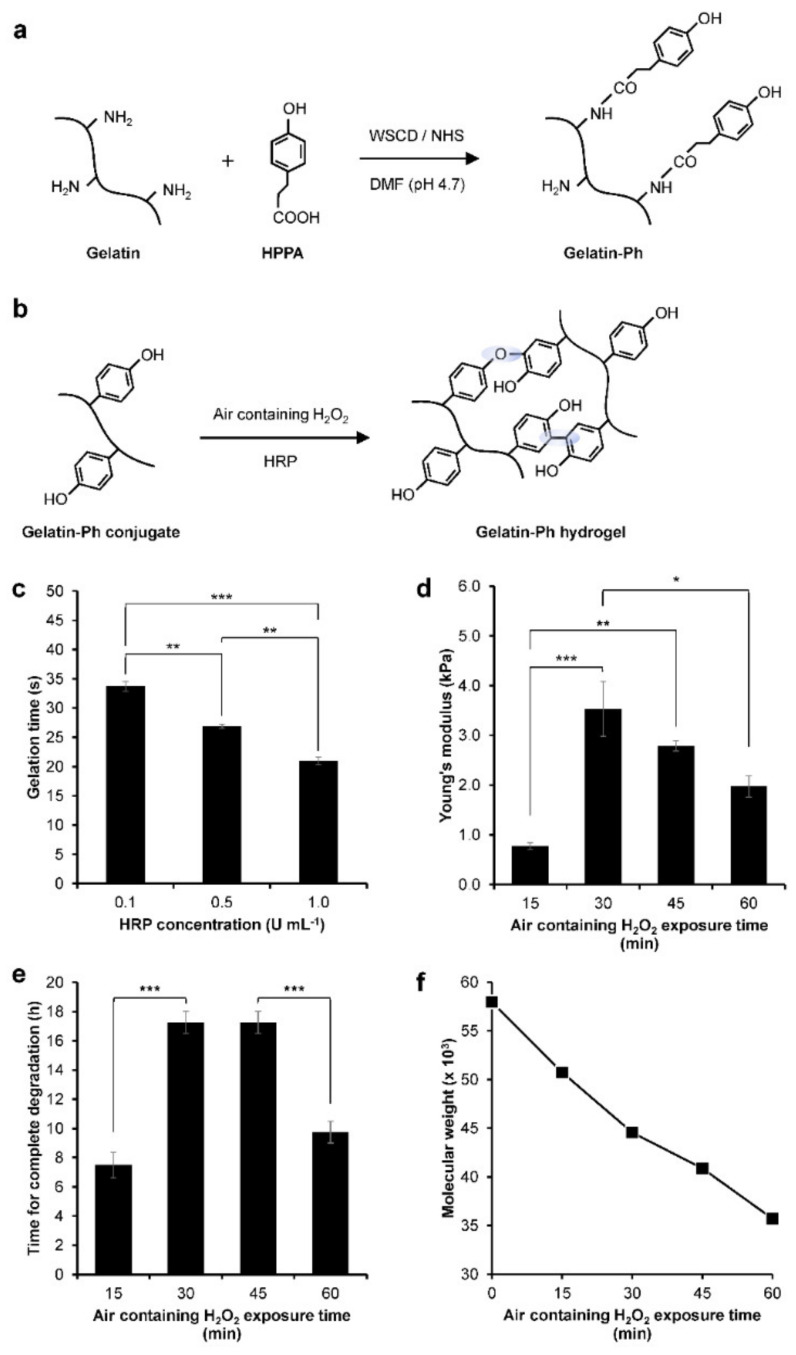
Characterisation of gelatin derivatives containing phenol groups (Gelatin-Ph) hydrogel. (**a**) Schematic of Gelatin-Ph preparation by conjugating gelatin with 3-(4-hydroxyphenyl) propionic acid (HPPA) using WSCD/NHS reaction in a DMF buffer (pH 4.7). (**b**) Schematic of HRP-catalysed cross-linking. (**c**) Gelation time of Gelatin-Ph hydrogel. Bar: S.E. (*n* = 3). (**d**) Young’s modulus of Gelatin-Ph hydrogel obtained through exposing 16 ppm air containing H_2_O_2_ for 15–60 min. Bar: S.E. (*n* = 5). (**e**) Degradability of Gelatin-Ph hydrogel by collagenase. Bar: S.E. (*n* = 4). (**f**) Molecular weight of Gelatin-Ph exposed with air containing H_2_O_2_ for 0–60 min. * *p* < 0.05, ** *p* < 0.005, *** *p* < 0.0005, Tukey’s HSD.

Gelatin-Ph was successfully prepared by conjugating the gelatin with HPPA via WSCD/NHS chemistry in a DMF buffer pH 4.7 (Figure 2a). UV-Vis observation showed the peak at 275 nm corresponding to the phenol (Ph) group (Figure S1). The Ph-content was measured at 2.3 × 10^−4^ mol-Ph g-Gelatin-Ph^−1^ based on the tyramine standard. Next, we investigated the hydrogelation of Gelatin-Ph. In this study, hydrogelation is induced by HRP-catalysed cross-linking in the presence of air containing H_2_O_2_ (Figure 2b). Exposing air containing 16 ppm H_2_O_2_ to a PBS solution containing 3.0% *w*/*v* Gelatin-Ph and 0.1, 0.5, and 1.0 U mL^−1^ HRP resulted in hydrogel formation within 35 s. Additionally, the increase in the HRP concentration resulted in a shorter gelation time (Figure 2c). The shorter gelation time could be mediated by the higher phenolic radical generation in the higher HRP concentration [16].

The effect of air containing H_2_O_2_ exposure time on the properties of the Gelatin-Ph hydrogel was then investigated. The hydrogel was fabricated by exposing a PBS solution containing 3.0% *w*/*v* Gelatin-Ph and 1 U mL^−1^ HRP with air containing H_2_O_2_ for 15, 30, 45, and 60 min. We selected these parameters considering the ease of handling in room temperature. At 5.0% *w*/*v*, the Gelatin-Ph solution quickly forms hydrogel at room temperature, while at 1.0% *w*/*v*, the resultant hydrogels are too weak to handle for experiments. In addition, 1 U mL^−1^ HRP is used, since previous studies have reported an H_2_O_2_-mediated dynamic of the stiffening and softening of the hydrogels using a similar setup [15,17]. The scanning electron microscope (SEM) observation on the cross-section of the hydrogels showed a porous structure with a pore diameter of 28–42 µm (Figure S2). The mechanical property of the hydrogel was investigated by measuring the Young’s modulus. The Young’s modulus of the Gelatin-Ph hydrogels increased as the exposure time was extended from 0.77 ± 0.07 kPa at 15 min and peaked at 30 min at 3.53 ± 0.55 kPa. Further extending the exposure time to 45 and 60 min led to gradual decreases in the Young’s modulus of the hydrogel to 2.79 ± 0.10 kPa and 1.97 ± 0.21 kPa, respectively (Figure 2d). The enzymatic degradation by collagenase showed a shorter time for the complete degradation on softer hydrogel obtained from 15 and 30 min of the exposure (Figure 2e).

The dynamic trend in the mechanical property of the hydrogel that shows an initial increase, which peaked at 30 min, followed by a reduction in the Young’s modulus in prolonged exposure time (Figure 2d) is consistent with our previous studies on Gelatin-Ph hydrogel and Gelatin-Ph/HA-Ph composite hydrogel [15,17]. The decreased Young’s moduli of Gelatin-Ph hydrogel in prolonged exposure to air containing 16 ppm H_2_O_2_ could be a consequence of cross-linking inhibition due to HRP inactivation. The prolonged exposure increased the concentration of H_2_O_2_ that generated excess phenoxy radicals. The attack of these excess radicals induces side reactions in the peroxidase catalytic cycle that inhibit cross-linking [18,19,20,21]. Further inactivation in higher H_2_O_2_ could also have occurred due to HRP denaturation [20].

Additionally, the decreasing Young’s modulus at 45–60 min exposure time could also be caused by the degradation of Gelatin-Ph by H_2_O_2_. Molecular weight measurements showed a decreasing molecular weight of Gelatin-Ph following exposure with air containing H_2_O_2_ for 15–60 min (Figure 2f and Figure S3), demonstrating the Gelatin-Ph degradation in extended H_2_O_2_ exposure. H_2_O_2_ produced free radicals such as H·, O·, and OH· that could induce cleavage to degrade the polymer [22]. Indeed, previous studies have reported that H_2_O_2_ could degrade a variety of materials, including gelatin, via oxidation [23,24,25].

### 2.2. Myoblasts Viability

Cell-substrate interaction is important in the formation of muscle cells (myogenesis) during embryonic development and post-injury. In addition, understanding the effect of the physicochemical properties of the microenvironment is also important for studying muscle regeneration in vitro, which provides a crucial foundation for developing functional artificial tissues and in vivo or translational studies [3,4]. Therefore, we investigated the effect of the Young’s moduli and molecular weight changes of the Gelatin-Ph hydrogel by air containing H_2_O_2_ to regulate myoblasts’ behaviour.

**Figure 3:**
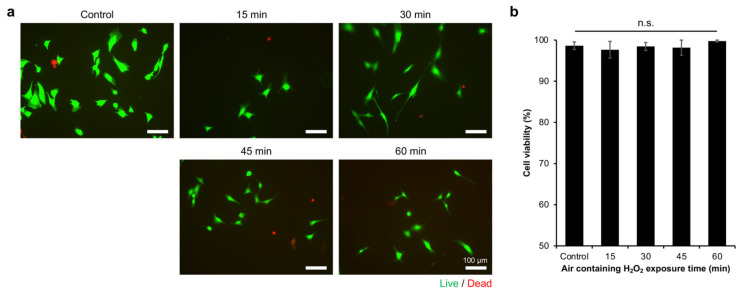
The viability of C2C12 myoblasts on Gelatin-Ph hydrogels obtained through varying air containing H_2_O_2_ exposure times. (**a**) Fluorescence micrograph of C2C12 myoblasts on day 2 of culture stained with Calcein-AM and propidium iodide (PI), which stained live and dead cells, respectively. (**b**) Viability of C2C12 myoblasts on the Gelatin-Ph hydrogels. Cells cultured on the culture well plate were used as the control. The data are presented as the means ± S.E. (*n* = 6). n.s.: *p* > 0.05, Tukey’s HSD.

In this study, we used C2C12 cells as the well-established myoblast cell line, which has been widely used as a skeletal muscle model [13,26,27,28]. Initially, we confirmed that the cells could attach to the hydrogel (Figure S4). Then, we investigated the viability of C2C12 myoblasts on the Gelatin-Ph hydrogels. The viability was analysed based on Calcein-AM/propidium iodide (PI) staining, which stained live and dead cells, respectively (Figure 3a). C2C12 cells showed the high viability (>94%) of the cells, independent of the exposure time to air containing H_2_O_2_ (Figure 3b). This result showed that, while the air containing H_2_O_2_ used in this study might intuitively be thought to induce cell death, the removal by catalase is sufficient to minimise or remove the toxic effect on cells. Additionally, the high viability of the cells could also be mediated by the well-known biocompatibility of Gelatin-Ph [29,30].

### 2.3. Myoblasts Adhesion

**Figure 4:**
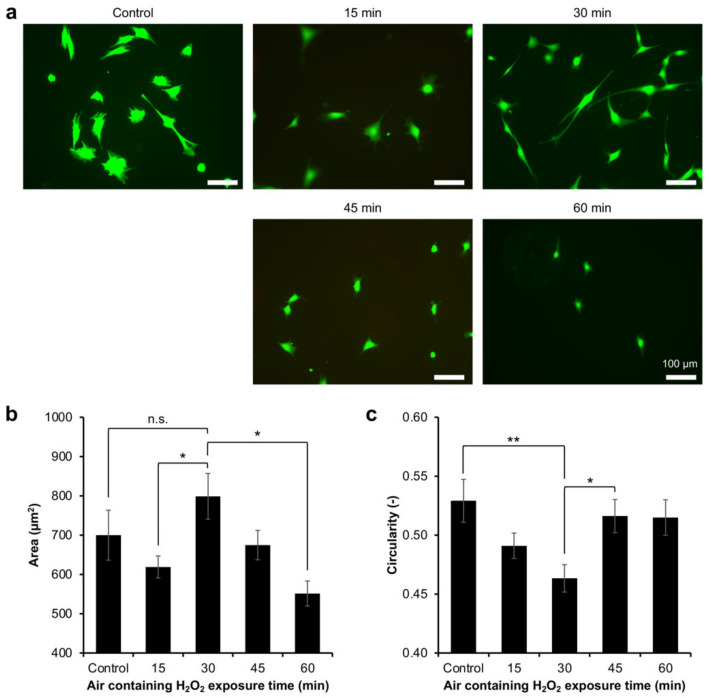
Adhesion of C2C12 myoblasts on Gelatin-Ph hydrogels obtained through exposure to air containing 16 ppm H_2_O_2_ for 15 to 60 min. (**a**) Fluorescence observation of C2C12 myoblasts stained with Calcein-AM cultured on the hydrogel for 2 days. As the control, the cells were cultured on the culture well plate. (**b**) Cell area and (**c**) circularity of C2C12 cells cultured on the resultant hydrogel. The data are presented as the means ± S.E. (*n* ≥ 114 cells). N.s.: *p* > 0.05, * *p* < 0.05, ** *p* < 0.005, Tukey’s HSD.

Based on the Calcein-AM staining during the viability analysis, it was observed that the cells had different morphologies on the hydrogels (Figure 3a). Previous studies have also reported that the Calcein, which stained the cytoplasm, allows for the observation of the cell morphology [31,32]. The difference in cell morphology could reflect the cell adhesion on the hydrogel. Therefore, the adhesion of myoblasts was investigated by analysing the morphology of the Calcein-AM-stained cells on the resultant Gelatin-Ph hydrogel. The myoblasts showed different morphologies on the Gelatin-Ph hydrogels obtained through different H_2_O_2_ exposure times (Figure 4a). The cells cultured on the hydrogel obtained through 30 min of the exposure, which has the highest mechanical property, had a large and elongated morphology, as shown by the largest cell area (Figure 4b) and lowest cell circularity (Figure 4c). In contrast, the cells cultured on the hydrogels obtained through air containing H_2_O_2_ exposure times of 15 min and 60 min had a more circular morphology, as shown by the lower cell area and higher circularity.

These results show that the adhesion of myoblasts depends on the stiffness of the Gelatin-Ph hydrogel. This phenomenon is similar to previous studies that cultured human mesenchymal stem cells, human adipose-derived stem cells, and fibroblasts on Gelatin-Ph hydrogel [15,33]. Additionally, smooth muscle cells and myoblasts also showed stiffness-dependent cell spreading on collagen-coated polyacrylamide gels and alginate hydrogel [34,35,36]. For adherent cells, including myoblasts, the adhesion dynamic depends on actin polymerisation and tension. A stiffer substrate allows actin polymerisation and assembly to occur and the cells to maintain the cytoskeletal tension, which results in the cell elongation [37,38]. In contrast, the cells cultured on the soft substrate cannot form F-actin bundles and stress fibres; thus, the cells appear in a round morphology [17,39].

Interestingly, we also found that the cells cultured on Gelatin-Ph hydrogel obtained through 30 min of exposure time have a more elongated shape than the cells on the plastic surface of the culture well plate, as observed from the significantly lower circularity (*p* < 0.005, Tukey’s HSD) (Figure 4c). The stiffness of the culture well plate is much higher (~1 GPa) [40], than that of the Gelatin-Ph hydrogel (0.77–3.53 kPa). Therefore, despite having a lower stiffness, the cell-adhesive property of Gelatin-Ph could be beneficial to controlling the elongation of the myoblasts. This phenomenon is possibly mediated by the focal adhesion kinase (FAK), the regulator of the cell elongation that is activated by the interaction between the Arg-Gly-Asp (RGD) tripeptide of the Gelatin-Ph and the integrins [41].

### 2.4. Myoblasts Differentiation

**Figure 5:**
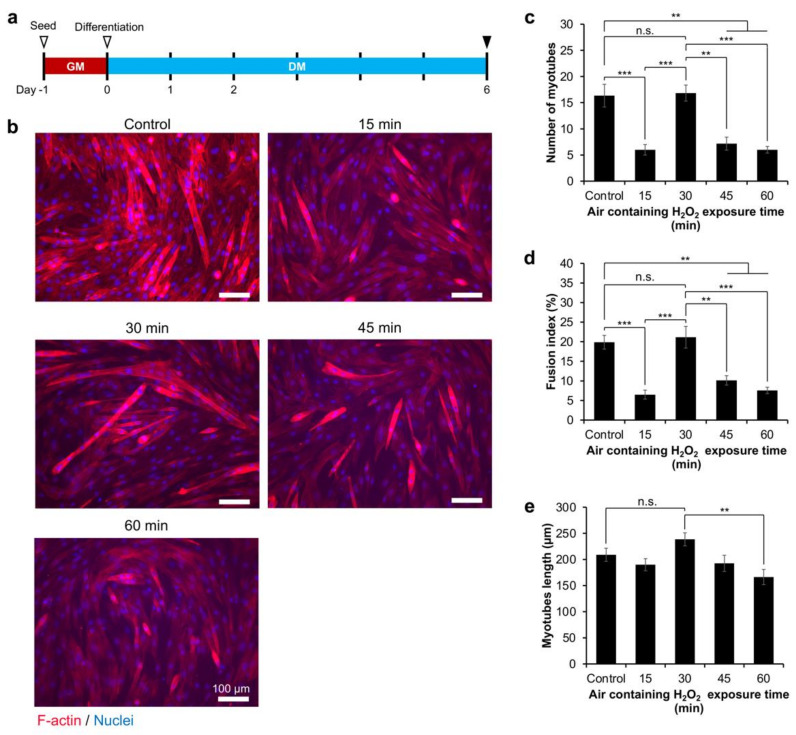
The differentiation of myoblasts to myotubes on the Gelatin-Ph hydrogel fabricated by altering the exposure time to air containing 16 ppm H_2_O_2_ for 15–60 min. (**a**) Experimental setup for inducing differentiation. After seeding, the cells were cultured in a growth medium (GM: DMEM + 10% fetal bovine serum). After 1 day in the GM, the medium was changed to a differentiation medium (DM: DMEM + 2% horse serum). (**b**) Fluorescence observation of C2C12 cells after 6 days in the differentiation medium stained with phalloidin and DAPI, staining F-actin and nuclei, respectively. (**c**) Number of myotubes (*n* = 6), (**d**) fusion index (*n* = 6), and (**e**) myotubes length (*n* ≥ 24). Bar: S.E. n.s.: *p* > 0.05, ** *p* < 0.005, *** *p* < 0.0005, Tukey’s HSD.

Finally, we investigated myoblasts’ differentiation into myotubes on the Gelatin-Ph hydrogels obtained from different exposures to H_2_O_2_. First, we seeded the cells on the culture well plate (control) and Gelatin-Ph hydrogels. After 1 day in the growth medium (DMEM + 10% fetal bovine serum), the cells’ density had reached > 80% confluency. Therefore, the medium was changed to a differentiation medium consisting of DMEM supplied with 2% horse serum (Figure 5a). After 6 days, the myotube formation was determined by observing the multinucleated cells, in which the F-actin and nuclei of the cells were stained with Phalloidin and DAPI, respectively.

The fluorescence observations showed different myoblasts differentiation trends based on air containing H_2_O_2_ exposure times to fabricate Gelatin-Ph hydrogel (Figure 5b). The cells cultured on the Gelatin-Ph hydrogel obtained through 30 min of H_2_O_2_ exposure showed the highest myotube formation, similar to the control on the culture well plate, as shown by the highest number of myotubes (Figure 5c) and the fusion index (Figure 5d). In contrast, on the hydrogels fabricated through 15, 45, and 60 min of exposure to air containing H_2_O_2_, C2C12 showed a significantly lower number of myotubes (Figure 5c) and a lower fusion index (Figure 5d). Furthermore, the myotubes cultured on hydrogel obtained through 30 min of the exposure also showed the longest myotube lengths (Figure 5e). These results showed that the myotube formation is also governed by the stiffness of the Gelatin-Ph hydrogel. 

In this study, the myogenesis was studied on hydrogel with a stiffness range of 0.77–3.53 kPa. This stiffness range is lower than that in previous studies, in which Tomasch et al. used 5–20 kPa fibrin hydrogels [42], Boonen et al. used Matrigel-coated polyacrylamide gels with a stiffness of 3–80 kPa [43], and Romanazzo et al. used 0.9–133.2 MPa poly-ε-caprolactone film [44]. However, the stiffness of the Gelatin-Ph hydrogel in this study is within the range of the reported stiffnesses of an intact (~0.5 kPa) and damaged skeletal muscle tissue (2–5 kPa) [45,46,47]. The stiffness-dependent myotube formation observed in this study is in accordance with previous reports [42,43,44]. A possible explanation for the higher myogenesis in the stiffness of 3.53 kPa than that in the stiffness of 0.77 kPa in this study is that, similar to the native skeletal muscle tissue [45], higher myoblast proliferation and differentiation are observed in damaged tissue compared to those in intact tissue. A similar conclusion was also reported by Trensz et al., who modelled intact and damaged tissue stiffness using polyacrylamide gels [47]. Mechanically, the lower myogenesis on the softer substrate could also be explained by the deformation or collapse of the substrate under cell contraction forces, which inhibits the myotubes formation [48].

Taken together, our study demonstrates that the contradictory effect of H_2_O_2_ on inducing cross-linking and degrading the polymer of the Gelatin-Ph hydrogel could modulate the adhesion and differentiation of myoblasts. However, future studies should be conducted to address the limitations of our current study. In the future, myogenesis studies on higher stiffness that better reflect the optimum stiffness to induce myogenesis (~12 kPa) should be conducted [34]. Myogenesis analysis based on specific markers such as MyoD or MF20 also should be used to further confirm the myoblasts differentiation. More importantly, the intricate details of the mechanotransduction of the myoblasts in response to the physicochemical changes of the Gelatin-Ph hydrogel also need to be studied. The interaction between the RGD sequence of the gelatin and the myoblasts receptor, e.g., the integrins, could be the key regulator [49,50,51]. Integrins could affect the YAP/TAZ pathway, which is reported to play a role in cell adhesion [39,52] and differentiation [53].

Additionally, there is a possibility that the physicochemical changes in the Gelatin-Ph hydrogel also affect the cell–cell communication that triggers myoblast fusion to form myotubes. During myogenesis, myotubes are formed by the fusion between myoblasts or the myoblast-myotube. Hindi et al. reported that the myoblast fusion could be mediated by integrins that increase the expression of the β1D integrin and caveolin-3 via focal adhesion kinase (FAK) [54]. Alternatively, a gelatin-based scaffold could also regulate the Intercellular Adhesion Molecule-1 (ICAM-1) [55] that plays role in myoblast fusion [56,57]. Further studies should be conducted to address the details of these pathways. We believe that our findings are useful in the field of biomedical engineering aimed at the regeneration of muscular tissue, which requires the knowledge of cell-substrate interaction [3]. Additionally, our findings could also be applied in the development of scaffolds with tuneable physicochemical and biological properties for biomedical applications [58,59].

## 3. Conclusions

The modulatory effect of H_2_O_2_ in HRP-catalysed cross-linking and the polymer degradation of the Gelatin-Ph hydrogel on controlling the myogenesis is reported. The myoblasts showed a high viability on the Gelatin-Ph hydrogel. The myoblasts showed a stiffness-dependent adhesion and differentiation, with higher elongation and myotube formation observed in higher stiffnesses. Taken together, these results showed that the H_2_O_2_-mediated changes in the properties of the Gelatin-Ph could govern the myogenesis. We believe that our findings could be useful for skeletal muscle tissue engineering to control the cell fate to form new muscle cells.

## 4. Materials and Methods

### 4.1. Materials

Gelatin from bovine (type B, ~226 g Bloom) was purchased from Sigma-Aldrich (St. Louis, MO, USA). *N*-hydroxysuccinimide (NHS), *N*,*N*-Dimethylformamide (DMF), 3-(4-hydroxyphenyl) propionic acid (HPPA), aqueous hydrogen peroxide (H_2_O_2_, 31% *w*/*w*), horseradish peroxidase (HRP, 190 U mg^−1^), catalase (bovine liver), collagenase, and 4% *w*/*v* paraformaldehyde in PBS were purchased from FUJIFILM Wako Pure Chemical (Osaka, Japan). Water-soluble carbodiimide hydrochloride (WSCD·HCl) was purchased from the Peptide Institute (Osaka, Japan). Dulbecco’s Modified Eagle Medium (DMEM) was purchased from Nissui (Tokyo, Japan). Calcein-AM was purchased from Nacalai Tesque Inc., Kyoto, Japan). Propidium iodide (PI) was purchased from Dojindo, Kumamoto, Japan). Phalloidin-iFluor 647 Reagent (ab176759) was purchased from Abcam (Cambridge, UK), and -Cellstain^®^- DAPI solution was obtained from Dojindo (Kumamoto, Japan).

### 4.2. Gelatin-Ph Preparation

Gelatin-Ph was prepared based on the previously reported methods [60,61]. Briefly, the gelatin was conjugated with HPPA in the DMF buffer (pH 4.7) via WSCD/NHS chemistry. After 20 h, the solution was dialysed in dH_2_O to remove the remaining HPPA, followed by freeze-drying. The presence of the Ph group introduced to the Gelatin-Ph was observed based on the peak at 275 nm using a UV-Vis spectrometer (UV-2600, Shimadzu, Kyoto, Japan).

### 4.3. Scanning Electron Microscope Observation

An aqueous solution containing 3.0% *w*/*v* Gelatin-Ph and 1 U mL^−1^ HRP in PBS was added to a polydimethylsiloxane (PDMS) mould (diameter: 8 mm, height: 4 mm). Air containing H_2_O_2_ was then exposed for 15, 30, 45, and 60 min. The resultant hydrogel was then frozen at −80 °C, immersed in 70% and 100% ethanol, and vacuum dried. The porous structure of the hydrogel was then observed using a scanning electron microscope (SEM, JCM-6000 plus, JEOL, Tokyo, Japan). The pore size was measured using ImageJ (1.53f51, NIH, Bethesda, MD, USA).

### 4.4. Gelation Time Measurement

The gelation time was measured based on previous reports [15,62]. A phosphate-buffered solution (PBS, pH 7.4) containing 3.0% *w*/*v* Gelatin-Ph and 0.1, 0.5, and 1 U mL^−1^ HRP was added to a well of a 48-well plate at 200 µL well^−1^. Air containing H_2_O_2_ (16 ppm), which was prepared by blowing air into 1 M H_2_O_2_ solution, was then exposed to the polymer solution, which was continuously stirred with a magnetic stirrer. Gel formation was indicated by the swelling of the surface and the hindrance of the magnetic stirrer.

### 4.5. Mechanical Property Measurement

Air containing H_2_O_2_ was exposed for 15–60 min to 600 µL PBS solution containing 3.0% *w*/*v* Gelatin-Ph and 1 U mL^−1^ HRP in a 12-well plate. The Young’s modulus of the fabricated Gelatin-Ph hydrogel was measured using a material tester (EZ-SX, Shimadzu, Kyoto, Japan). The hydrogels were compressed with a probe (ø: 8 mm) at a compression rate of 6.0 mm min^−1^. The Young’s modulus was calculated based on the stress–strain curve with a compression strain of 1–3% (Figure S5).

### 4.6. Enzymatic Degradation

The Gelatin-Ph hydrogels were immersed in the PBS solution for 24 h in order to reach an equilibrium state. The PBS solution was then changed to PBS containing 120 µg mL^−1^ collagenase. The time for the complete degradation of the hydrogel was observed using OLYMPUS Provi CM20 (Olympus, Tokyo, Japan).

### 4.7. Molecular Weight Measurement

The PBS solution containing 3.0% *w*/*v* Gelatin-Ph was exposed with air containing H_2_O_2_ for 0, 15, 30, 45, and 60 min. The molecular weights of the Gelatin-Ph were then measured using HPLC (LC-20AD, Shimadzu, Kyoto, Japan) and an RI detector (RID-20A, Shimadzu, Kyoto, Japan).

### 4.8. Cell Culture

The mouse muscle myoblasts C2C12 cell line was obtained from the RIKEN Cell Bank (Ibaraki, Japan). The C2C12 was cultured in a growth medium consisting of low glucose DMEM supplied with 10% *v*/*v* fetal bovine serum (FBS). The cells were cultured at a 37 °C incubator supplied with 5% CO_2_.

### 4.9. Cell Viability and Adhesion Analysis

The hydrogel was fabricated by exposing 600 µL well^−1^ PBS solution containing 3.0% *w*/*v* Gelatin-Ph and 1 U mL^−1^ HRP to air containing H_2_O_2_ for 15–60 min. One millilitre of the growth medium containing 1 mg mL^−1^ catalase was added to the hydrogel to remove the remaining H_2_O_2_. After overnight incubation in the medium containing catalase, the hydrogels were washed with the PBS and growth medium. The C2C12 cells were then seeded on the hydrogel or culture well plate as the control at 3.6 × 10^3^ cells cm^−2^. The viability of the cells was observed after 2 days of culture by staining the cells with 3.3 µg mL^−1^ Calcein-AM/3.3 µg mL^−1^ propidium iodide (PI) in the PBS for 10 min, which stained the viable and dead cells, respectively. The Calcein-AM/PI-stained cells were observed using a fluorescence microscope (BZ-9000, Keyence, Osaka, Japan). The viability of the cells was calculated as the percentage of the number of viable cells/the total number of cells. Cell adhesion was analysed based on the morphological characteristics of the cells observed on day 2 of the culture. The cells’ morphological parameters, including the cell area and circularity, were measured using ImageJ. The circularity was calculated as 4π × (area/perimeter^2^).

### 4.10. Cell Differentiation Analysis

The C2C12 cells were seeded onto the culture well plate (control) and the Gelatin-Ph hydrogels at a density of 2.6 × 10^4^ cells cm^−2^. After one day, the growth medium was changed to a differentiation medium consisting of DMEM containing 2% *v*/*v* horse serum. The differentiation medium was replenished every two days, and the multinucleated myotubes were observed on day 6 post-induction [63,64]. The myotubes were observed by staining the cells with Phalloidin-iFluor 647 Reagent and -Cellstain^®^- DAPI solution. Briefly, the cells were fixed with 4% paraformaldehyde in the PBS for 30 min, permeabilised in 4-(2-hydroxyethyl)-1-piperazineethanesulfonic acid (HEPES) pH 5.5 for 10 min, and stained with Phalloidin (1×) in the PBS for 60 min and with DAPI (100 nM) in the PBS for 30 min. The number of myotubes and nuclei, as well as the myotube lengths, were analysed using ImageJ. The fusion index was calculated as the percentage of the number of nuclei in the myotubes/the total number of nuclei.

### 4.11. Statistical Analysis

The data were analysed using Microsoft^®^ Excel^®^ 2019 version 1808 (Microsoft Corp., Redmond, WA, USA). The statistical analysis was conducted using a one-way analysis of variance (ANOVA). A post hoc *t*-test was conducted using Tukey’s HSD; a *p*-value <0.05 was considered significantly different.

## Data Availability

All data generated or analysed during this study are included in this published article and its supplementary files.

## Supplementary Materials

The following supporting information can be downloaded at: https://www.mdpi.com/article/10.3390/gels8060387/s1, Figure S1: UV-Vis absorbance of the unmodified gelatin and Gelatin-Ph; Figure S2: (a) Scanning electron microscope (SEM) observation of the cross-section of the Gelatin-Ph hydrogel. (b) Pore size of the Gelatin-Ph hydrogel obtained through different air containing H_2_O_2_ exposure times; Figure S3: Intensity-time curve of the Gelatin-Ph exposed with air containing H_2_O_2_ for 0–60 min; Figure S4: Confocal laser-scanning microscope observation of the C2C12 myoblasts on the culture well plate (control) and the Gelatin-Ph hydrogel obtained through exposure to air containing H_2_O_2_ for 15, 30, 45, and 60 min.; Figure S5: Stress-strain curve of the Gelatin-Ph hydrogel fabricated by exposing the solution containing 3.0% *w*/*v* Gelatin-Ph and 1 U mL^−1^ HRP with air containing H_2_O_2_ for 15, 30, 45, and 60 min.
