# Supplementary material for: Tuning Myogenesis by Controlling Gelatin Hydrogel Properties through Hydrogen Peroxide-Mediated Cross-Linking and Degradation
Source: Gels. 2022 Jun 17;8(6):387. doi: 10.3390/gels8060387 (PMC9223222; doi:10.3390/gels8060387)
Supplement: Supplementary file 1 [file gels-08-00387-s001.zip › gels-1775786-supplementary.pdf]

gels

Supplementary Information

Tuning Myogenesis by Controlling Gelatin Hydrogel Properties through Hydrogen Peroxide-Mediated Cross-Linking and Degradation

Wildan Mubarok, Kelum Chamara Manoj Lakmal Elvitigala and Shinji Sakai *

Department of Materials Engineering Science, Graduate School of Engineering Science, Osaka University, Toyonaka 560-8531, Japan; wildanmubarok@cheng.es.osaka-u.ac.jp (W.M.); kelum@cheng.es.osaka-u.ac.jp (K.C.M.L.E.)
*Correspondence: sakai@cheng.es.osaka-u.ac.jp

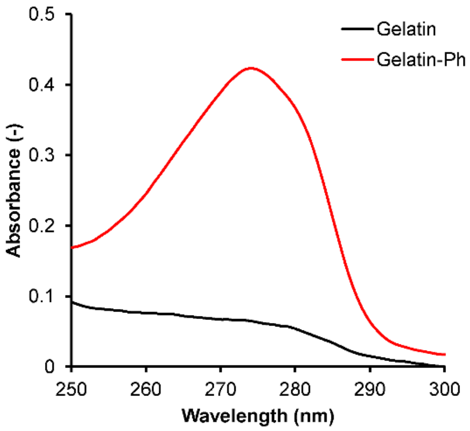

Figure S1. UV-Vis absorbance of unmodified gelatin and Gelatin-Ph. Notice the peak at 275 nm corresponding to the Ph group.

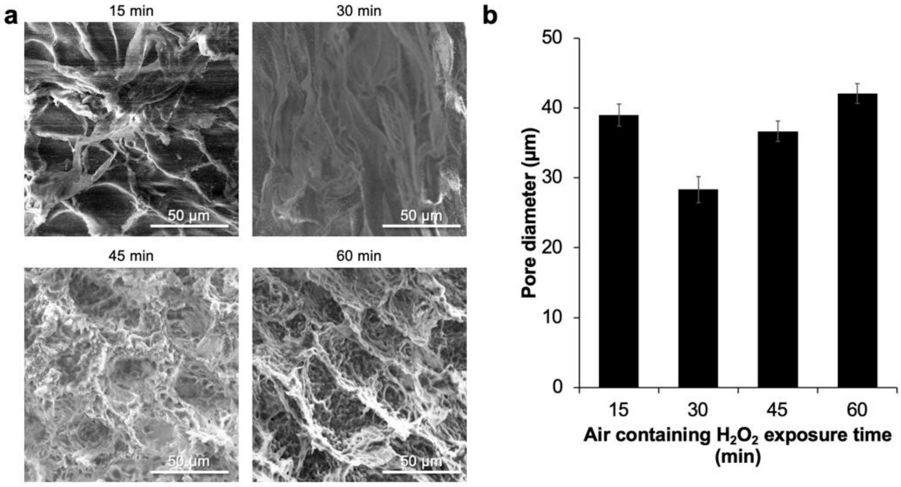

Figure S2. (a) Scanning electron microscope (SEM) observation of the cross-section of Gelatin-Ph hydrogel. (b) Pore size of the Gelatin-Ph hydrogel obtained through different air containing H_2O_2 exposure times. Bar: S.E. (n = 40).

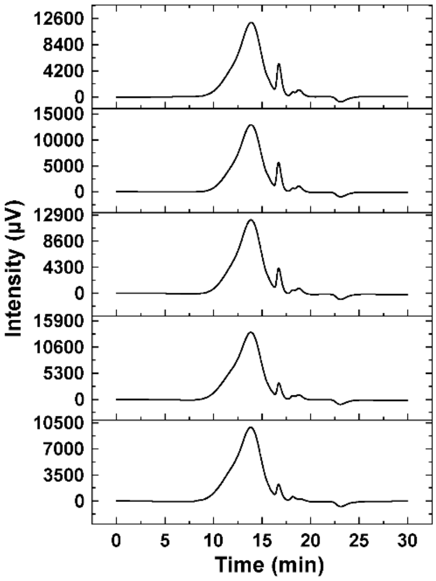

Figure S3. Intensity-time curve of the Gelatin-Ph exposed with air containing H_2O_2 for 0–60 min.

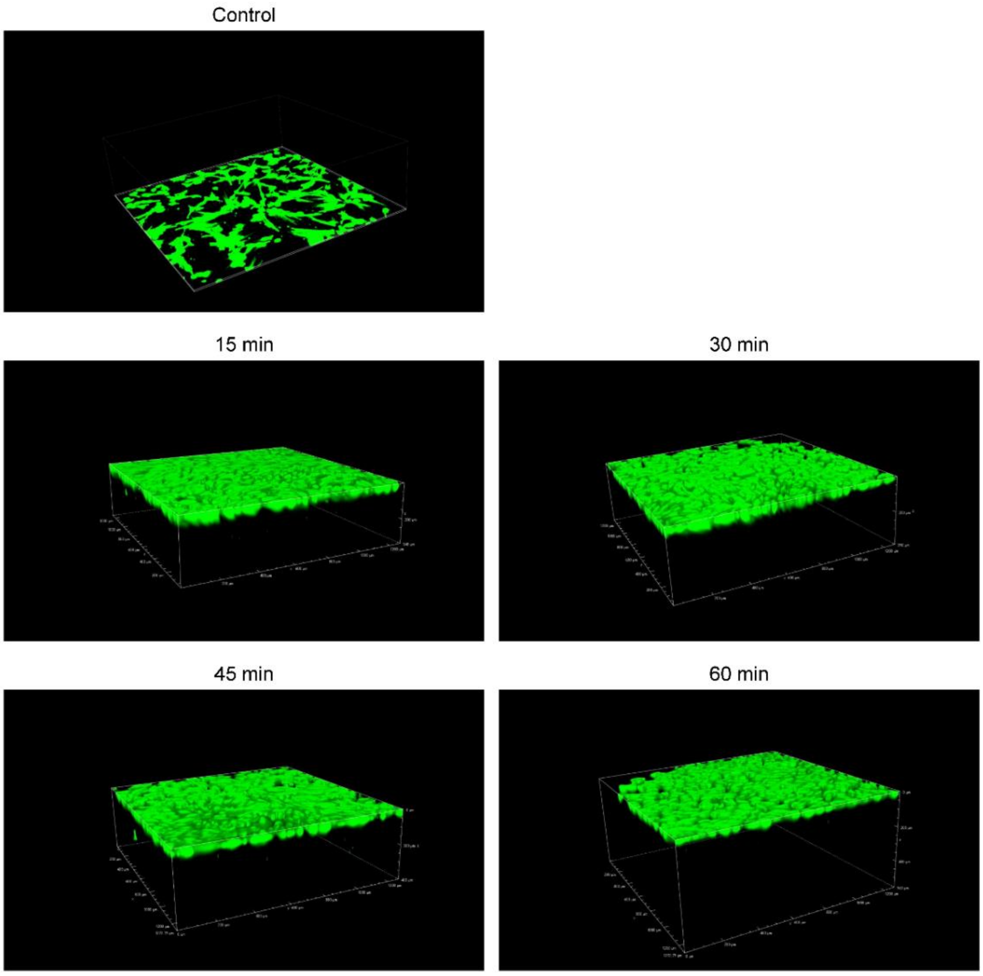

Figure S4. Confocal laser-scanning microscope observation of the C2C12 myoblasts on the culture well plate (control) and Gelatin-Ph hydrogel obtained through exposure to air containing H_2O_2 for 15, 30, 45 and 60 min. Cells were observed by staining with Calcein-AM on day 2 of culture.

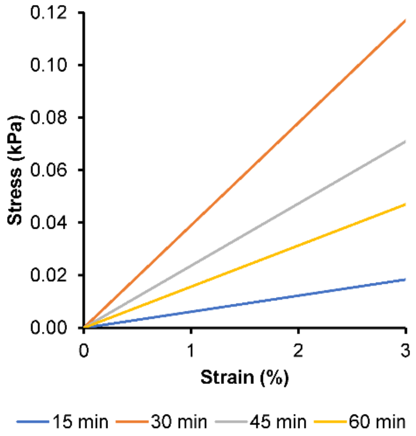

Figure S5. Stress-strain curve of Gelatin-Ph hydrogel fabricated by exposing solution containing 3.0% w/v Gelatin-Ph and 1 U mL^{-1} HRP with air containing H_2O_2 for 15, 30, 45 and 60 min.
